# Deep Learning-Assisted 3D Analysis of Coronoid Process Changes After Orthognathic Surgery

**DOI:** 10.3390/jcm15134939

**Published:** 2026-06-25

**Authors:** Jacek Rożko, Paweł Piotr Grab, Michał Szałwiński, Dominika Zawadka-Modras, Maria Sobol, Bartosz Startek, Dariusz Jurkiewicz, Aldona Chloupek

**Affiliations:** 1 Department of Cranio-Maxillo-Facial Surgery, Military Institute of Medicine—National Research Institute, Szaserów 128, 04-141 Warsaw, Poland; 2 Department of Biophysics, Physiology and Pathophysiology, Medical University of Warsaw, Chałubińskiego 5, 02-004 Warsaw, Poland; 3 Stardent—Private Surgical Practice, ul. Fryderyka Chopina 3/1, 59-700 Bolesławiec, Poland; 4 Department of Otolaryngology, Military Institute of Medicine—National Research Institute, Szaserów 128, 04-141 Warsaw, Poland

**Keywords:** orthognathic surgery, deep learning, three-dimensional analysis, automatic segmentation, surface-based registration

## Abstract

**Background/Objectives:** Postoperative remodeling and positional deviations of the mandibular coronoid process (CP) after orthognathic surgery remain insufficiently characterized, particularly in three-dimensional analyses. The aim of this study was to evaluate qualitative and quantitative CP changes following bimaxillary orthognathic surgery using a deep learning-assisted three-dimensional workflow. **Methods:** This retrospective study included 75 patients treated with combined orthodontic–surgical therapy, including 25 patients with skeletal Class II malocclusion and 50 patients with skeletal Class III malocclusion. Preoperative and 6-month postoperative computed tomography scans were analyzed. Automatic segmentation and three-dimensional reconstruction were performed using a convolutional neural network based on the nnU-Net architecture. Qualitative assessment included evaluation of CP displacement patterns and visualization of local surface differences using heat maps. Quantitative analysis included volumetric assessment of preoperative and postoperative CP models, calculation of apposition-compatible (Vapo) and resorption-compatible (Vres) volumetric changes, and mixed-effects modeling accounting for within-patient correlations. **Results:** Medial displacement of the CP predominated in both skeletal classes and was more frequent in Class III patients. Qualitative surface analysis demonstrated a consistent location-dependent remodeling pattern, characterized by predominant apposition-compatible changes on the lateral and medial surfaces and predominant resorption-compatible changes along the anterior border. Quantitative analyses revealed an overall positive remodeling balance, although substantial inter-individual variability was observed. Mixed-effects analyses demonstrated no significant overall effects of side or skeletal class on volumetric remodeling; however, a significant interaction between side and skeletal class was identified for net remodeling balance. A significant random patient effect indicated considerable variability in remodeling response among individuals. **Conclusions:** AI-assisted three-dimensional analysis enables a reproducible assessment of postoperative CP remodeling following orthognathic surgery. Coronoid process remodeling is characterized by heterogeneous, location-dependent surface changes and substantial inter-individual variability. The observed remodeling patterns are compatible with adaptive responses to altered postoperative biomechanical conditions, although the underlying biological mechanisms remain to be clarified.

## 1. Introduction

Orthognathic surgery induces substantial changes in the spatial relationships of craniofacial skeletal structures and associated soft tissues. These changes trigger adaptive responses within the stomatognathic system. Understanding postoperative changes is important for evaluating treatment outcomes, long-term stability, and biological adaptation.

Orthodontic–surgical treatment of skeletal malocclusions results in remodeling of the osseous structures of the craniofacial complex [1]. Alteration of functional conditions following orthognathic surgery suggests an influence on the temporalis muscle (TM), which plays a key role in masticatory biomechanics. The coronoid process (CP), as the insertion site of this muscle, may potentially exhibit an adaptive response. Changes within the CP include both volumetric alterations and deviations from its original position. This phenomenon is most likely associated with the repositioning of the proximal segment achieved through linear and angular movements performed during bilateral sagittal split osteotomy (BSSO). These surgical modifications may alter the tension and functional loading of the TM, potentially contributing to adaptive changes at its insertion site [2]. This mechanism is consistent with the concept of adaptive bone remodeling in response to mechanical loading (Wolff’s law) [3].

Advances in three-dimensional imaging and deep learning-based tools have enabled increasingly precise quantitative and qualitative assessment of craniofacial changes. Automated segmentation and 3D reconstruction performed by trained neural networks allow for accurate comparison of anatomical structures before and after surgery. The use of artificial intelligence (AI) can reduce analysis time and improve efficiency while maintaining clinically acceptable accuracy compared with conventional manual techniques. However, the accuracy of such analyses remains dependent on the performance and validation of the underlying models [4].

Previous studies have primarily focused on remodeling of the mandibular condyle and general changes within the mandibular ramus, whereas data concerning the coronoid process—particularly in three-dimensional and volumetric analyses—remain limited. Although the mandibular condyle has traditionally attracted the most attention because of its role in postoperative stability and temporomandibular joint function, the coronoid process also represents a biologically relevant structure. As the insertion site of the temporalis muscle, it is subjected to altered biomechanical loading following orthognathic surgery and may therefore undergo adaptive remodeling. Despite this potential biological and clinical significance, postoperative changes in the coronoid process have rarely been investigated using three-dimensional quantitative methods. Consequently, the magnitude, direction, and surface distribution of postoperative coronoid process remodeling remain poorly understood.

### Aim of the Study

The aim of the present study was to investigate postoperative changes in the mandibular coronoid process following orthognathic surgery using a deep learning-assisted three-dimensional analysis workflow. Specifically, the study evaluated positional deviations, volumetric changes, and surface remodeling patterns of the coronoid process by comparing preoperative and postoperative 3D models. In addition, differences between skeletal Class II and Class III patients were assessed to determine whether the type of dentofacial deformity influences the magnitude and distribution of postoperative remodeling.

## 2. Materials and Methods

### 2.1. Patient Selection

This retrospective study included 75 patients:

A total of 25 with skeletal Class II malocclusion (∠ANB > 4°).

A total of 50 with skeletal Class III malocclusion (∠ANB < 0°).

The cohort comprised 54 women and 21 men aged 18–48 years, treated with combined surgical–orthodontic therapy between January 2023 and June 2025 (demographic characteristics of the study groups are presented in Supplementary Table S1). All patients underwent surgery at the Department of Cranio-Maxillofacial Surgery, Military Institute of Medicine—National Research Institute, Warsaw. Based on the study methodology, inclusion and exclusion criteria were defined.

Inclusion criteria: age ≥ 18 years, no previous orthognathic surgery, skeletal Class II or III, and preoperative orthodontic preparation with fixed appliances.

Exclusion criteria: age < 18 years, post-traumatic or postoperative changes in the temporomandibular joint region, non-compliance with follow-up visits, and failure to continue orthodontic treatment after surgery.

Patients presenting with facial asymmetry were not excluded from the study. The cohort was intended to reflect the spectrum of dentofacial deformities routinely treated with orthognathic surgery in clinical practice.

### 2.2. Treatment Protocol

All patients were treated according to a standardized surgical–orthodontic protocol. Preoperative orthodontic treatment aimed to prepare patients for surgery through dental decompensation. Surgical planning was performed using IPS Case Designer software (version 2.5.7; KLS Martin Group, Tuttlingen, Germany), with two surgical splints (intermediate and final) generated for each case and exported in stereolithography format (STL). The splints were 3D-printed (NextDent 5100; NextDent, Soesterberg, The Netherlands) using dedicated medical resin (NextDent SG; NextDent, Soesterberg, The Netherlands).

Bimaxillary (BiMax) orthognathic surgery using a mandible-based protocol was performed according to routine clinical practice by the same experienced surgical team. All patients underwent Le Fort I maxillary osteotomy and BSSO, performed with a piezoelectric saw and bone burs. Stable fixation was achieved using titanium miniplates of the KLS Martin 2.0 system (KLS Martin Group, Tuttlingen, Germany). The mandibular proximal segment was positioned manually, with the aim of establishing a centered condylar position within the temporomandibular joint.

### 2.3. Imaging Data

All radiological examinations were performed using a 64-slice CT scanner (Revolution EVO, GE Medical Systems, Chicago, IL, USA). Each patient underwent two scans: 2 weeks preoperatively (T0) and 6 months postoperatively (T1), both acquired in the supine position with natural occlusion. Slice thickness was 0.625 mm, while axial pixel spacing varied between series, ranging from 0.328 to 0.488 mm. The field of view (FOV) in the XY plane was 226 × 226 mm.

### 2.4. Data Standardization and Preprocessing

To standardize tomographic data, all volumes underwent spatial resolution (voxel size) harmonization to ensure comparability of pre- and postoperative scans. CT images in DICOM (Digital Imaging and Communications in Medicine) format were imported into 3D Slicer (version 5.8.1; www.slicer.org), where resampling to a common spatial grid was performed. The target voxel size was set to match the dataset with lower spatial resolution, using linear interpolation. For each patient, preoperative and postoperative datasets were resampled to a common voxel size corresponding to the lower spatial resolution of the two examinations. Consequently, voxel size was standardized within each patient to ensure comparability between T0 and T1 scans. Because the original acquisition parameters differed among patients, the final voxel size after harmonization still ranged from 0.328 × 0.328 mm to 0.488 × 0.488 mm across the study cohort, while remaining identical within each patient pair.

### 2.5. Analytical Software

The research workflow was conducted using proprietary software OrtogOnBlender Startek Edition (OOB-SE), an extension of the Blender environment (version 4.5.1; Blender Foundation, Amsterdam, The Netherlands) with dedicated clinical and analytical modules. The software integrates tomographic data processing, automatic segmentation of anatomical structures, generation of three-dimensional mesh models, and tools for model registration as well as qualitative and quantitative analyses within a single environment [5].

### 2.6. Data Analysis

For the analysis of radiological data, automated segmentation and three-dimensional reconstruction of computed tomography scans obtained at T0 and T1 were performed, generating preoperative (Mpre) and postoperative (Mpost) models. The analysis consisted of two stages: qualitative assessment of CP changes, including evaluation of deviation relative to the original position and identification of regional surface remodeling patterns, as well as quantitative assessment of volumetric changes in the CP.

#### 2.6.1. Automatic Segmentation and 3D Reconstruction

Automatic segmentation was performed within the OOB-SE environment using a pretrained deep learning model based on the nnU-Net architecture (3D full-resolution variant). Detailed information regarding the segmentation framework, network architecture, training procedure, and validation metrics is provided in the Supplementary Material.

The model enabled fully automatic extraction of anatomical structures from CT data, providing a standardized and reproducible segmentation workflow. In the present study, the coronoid process was not segmented as a separate anatomical class but formed part of the mandibular segmentation mask used for subsequent analyses.

Three-dimensional reconstruction of craniofacial models was performed automatically from the generated segmentation masks. The 3D meshes were created without manual modification of geometry, ensuring consistent conversion of volumetric radiological data into precise models in STL format, representing the morphology of the analyzed structures in both the preoperative and postoperative states.

Subsequently, an automated mesh-closing procedure was applied to generate watertight solid models suitable for volumetric analysis. This procedure was performed algorithmically after user initiation and did not involve manual editing of vertices, edges, or mesh surfaces.

#### 2.6.2. Qualitative Assessment of CP Deviation Relative to Its Original Position

**Figure 1:**
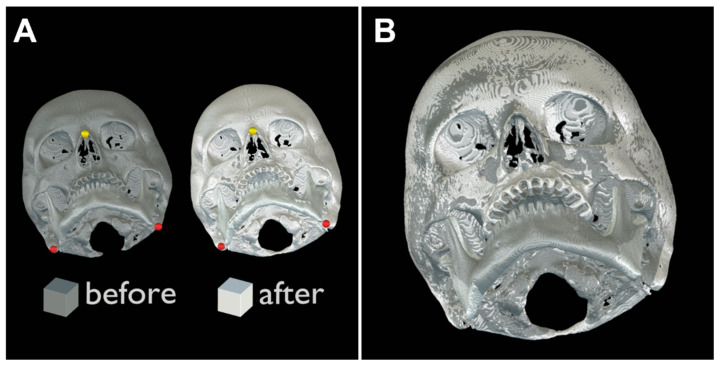
(**A**) Preoperative (gray) and postoperative (white) cranial models used for the initial orientation of the superimposition procedure. The yellow landmark indicates the rhinion, whereas the red landmarks indicate the bilateral mastoidale points. (**B**) Visualization of the models following initial superimposition based on the selected craniometric landmarks.

**Figure 2:**
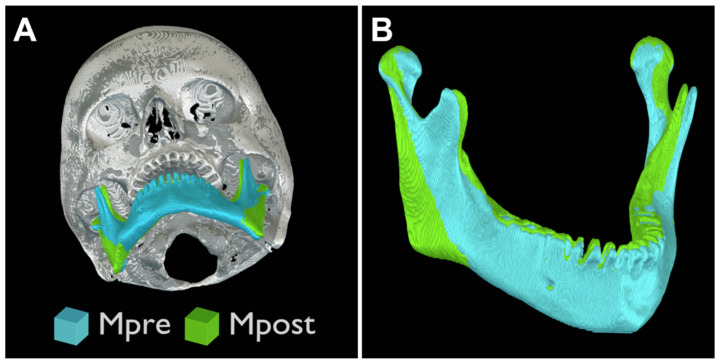
(**A**) Distinct colors were assigned to the superimposed mandibular models to facilitate visual differentiation between the preoperative and postoperative structures. (**B**) The mandibular models were isolated from the cranial structures to visualize medial and lateral deviation of the coronoid process (CP).

To compare the position of the coronoid process before and after surgery, initial model alignment was performed by manually selecting craniometric landmarks located in regions unaffected by surgery: the rhinion and bilateral mastoidale. These reference points were used to ensure correct spatial orientation of the preoperative (Mpre) and postoperative (Mpost) models relative to each other (Figure 1). Following alignment, a qualitative assessment was performed. To facilitate visual differentiation, distinct colors were assigned to the mandible models, which were isolated from the cranial structures in both Mpre and Mpost, allowing visualization of coronoid process deviation in either the medial or lateral direction (Figure 2). The direction of coronoid process displacement (medial or lateral) was assessed qualitatively based on visual inspection of superimposed preoperative and postoperative three-dimensional models following ICP registration. Classification was assigned according to the predominant direction of displacement observed across the coronoid process surface. No predefined quantitative displacement threshold was applied. All assessments were performed by a single examiner.

#### 2.6.3. Quantitative Assessment of Volumetric Changes

**Figure 3:**
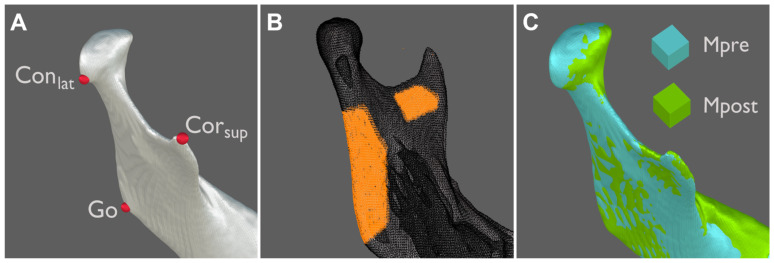
(**A**) Anatomical landmarks used for definition of the region of interest. (**B**) Registration mask (orange) comprising the posterior mandibular ramus above the gonial angle and below the condylar neck, together with the sub-coronoid region inferior to the coronoid process and mandibular notch. These regions were selected according to previously validated studies demonstrating their postoperative stability following BSSO. (**C**) Example of superimposed preoperative and postoperative models after ICP registration.

The second stage was performed separately for the right and left CPs using the pre- and postoperative mandibular models isolated in the previous step. As in the first stage, initial coarse alignment was achieved by manually selecting three pairs of reference landmarks located in easily identifiable anatomical structures of the mandible: gonion, condylion lateralis, and coronoid superius, corresponding to anatomical landmarks described in CBCT-based analyses [6]. Final superimposition was performed using ICP surface-based best-fit alignment, restricted to stable, unaffected reference regions of the mandibular ramus, which are considered stable following BSSO according to current literature [7] (Figure 3). The registration mask included the posterior aspect of the mandibular ramus located above the gonial angle and below the condylar neck, as well as the sub-coronoid region inferior to the coronoid process and mandibular notch. These regions were selected based on the stability analysis reported by Holte et al., who demonstrated that they exhibit minimal postoperative surface change following BSSO and therefore represent the most stable anatomical structures available for regional mandibular superimposition. The reference regions were predefined before analysis and applied uniformly to all cases.

This approach was intended to minimize the influence of global mandibular displacement and to allow assessment of local surface changes within the coronoid process. Nevertheless, because the coronoid process forms part of the proximal mandibular segment, the possibility that a component of the observed surface differences reflects residual positional changes, minor registration inaccuracies, segmentation uncertainty, or mesh-processing artifacts rather than exclusively biological remodeling cannot be completely excluded. Consequently, the terms “bone apposition” and “bone resorption” are used in the present study as descriptors of surface-based geometric changes identified after registration and should be interpreted within the methodological limitations of image-based shape analysis.

**Figure 4:**
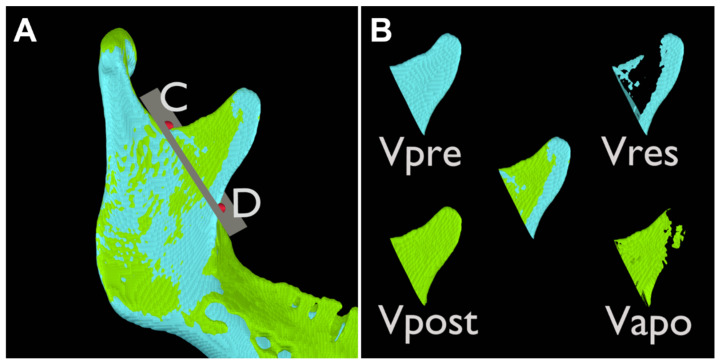
(**A**) Illustration of the plane passing through points C and D used to define the ROI. (**B**) Schematic representation of the quantitative volumetric analysis of the CP. After isolating the ROI, the volumes of the preoperative (Vpre) and postoperative (Vpost) models were calculated. Subsequently, Boolean operations were applied to determine imaging-derived appositional (Vapo; Vpost − Vpre) and resorptive (Vres; Vpre − Vpost) volumetric differences between the registered models, and their respective volumes were quantified.

For the quantitative analysis, a region of interest (ROI) encompassing the coronoid process was first isolated from the entire mandibular model. To ensure a consistent and reproducible cutoff level in each Mpre–Mpost pair, the ROI boundary was defined based on the coronoid process base plane (CP base plane), following the methodology described by He et al. [8]. This plane was defined as passing through point C (the deepest point of the mandibular notch) and point D (the posterior point of the coronoid process along its anterior border). After ROI extraction, CP volume was calculated for both preoperative (Vpre) and postoperative (Vpost) models. Volumetric differences compatible with resorptive remodeling (Vres) were obtained by subtracting Vpost from Vpre, whereas volumetric differences compatible with appositional remodeling (Vapo) were determined by subtracting Vpre from Vpost using Boolean operations (Figure 4).

Volumetric change was expressed as the relative volume difference according to the formula:

ΔV = (Vpost − Vpre)/Vpre.

Additionally, the relative proportion of volumetric differences compatible with resorptive and appositional remodeling was calculated as Vres/Vpre × 100 and Vapo/Vpre × 100, respectively.

#### 2.6.4. Qualitative Assessment of Local Surface Differences Within the Coronoid Process

**Figure 5:**
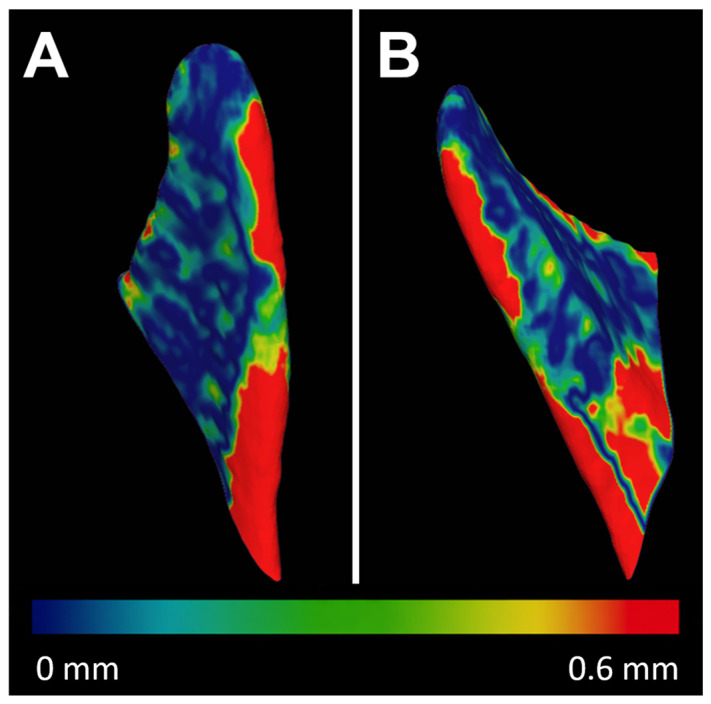
Representative heat maps illustrating local surface discrepancies between preoperative (Mpre) and postoperative (Mpost) coronoid process models following ICP-based registration. The color scale represents the magnitude of surface difference between the registered models (0–0.6 mm). Blue regions indicate minimal surface discrepancy, whereas red regions indicate greater surface discrepancy. The maps were used as a qualitative visualization tool and do not represent signed surface distances or a threshold-based classification of biological remodeling. (**A**) Lateral surface. (**B**) Medial surface.

The analysis was performed using the regions of interest (ROIs) isolated in the previous stage, encompassing the coronoid processes. Following superimposition of the preoperative and postoperative models, heat map analysis was performed to visualize the spatial distribution of local surface differences within the CP. The heat maps represented unsigned surface distances between the registered models and were displayed using a color scale ranging from 0 to 0.6 mm, where blue indicated regions of minimal surface discrepancy and red indicated regions of maximal discrepancy. Because the visualization was based on absolute surface distance, the heat maps did not directly indicate the direction of change (apposition or resorption). Instead, they were used as a qualitative visualization tool to identify regions exhibiting greater or lesser local surface discrepancy between the registered preoperative and postoperative models (Figure 5).

The qualitative classification of appositional and resorptive surface patterns was performed independently of the heat map visualization and was based on visual assessment of the spatial relationship between registered preoperative and postoperative models. Heat maps were used solely to illustrate the magnitude and distribution of local surface differences.

### 2.7. Statistical Analysis

Statistical analysis was performed using Statistica 13.3 software (TIBCO Software Inc., Palo Alto, CA, USA). Normality of data distribution was assessed with the Shapiro–Wilk test. For comparisons between preoperative and 6-month postoperative measurements, the paired Student’s *t*-test was applied when the assumption of normality was met, whereas the Wilcoxon signed-rank test was used otherwise. Exact *p*-values were reported for all analyses.

To evaluate the overall remodeling pattern, the net volumetric balance (Vnet) was calculated as the difference between Vapo and Vres (Vnet = Vapo − Vres), where both parameters represent imaging-derived volumetric differences compatible with appositional and resorptive remodeling patterns. Absolute and relative changes were calculated and reported together with 95% confidence intervals (95% CI).

Given the bilateral nature of the coronoid process measurements, additional mixed-effects models were applied to account for within-subject correlation while preserving side-specific analyses. Mixed-effects models were estimated with the patient included as a random effect and side and skeletal class included as fixed effects. Interaction terms (side × skeletal class) were also tested where appropriate. Degrees of freedom were estimated using the Satterthwaite approximation. Effect sizes for paired comparisons were quantified using Cohen’s dz.

The primary aim of this study was exploratory and hypothesis-generating rather than confirmatory. Therefore, formal adjustment for multiple comparisons was not applied.

All statistical analyses were two-sided, and the level of statistical significance was set at α = 0.05.

### 2.8. Intra-Observer Reproducibility and Registration Accuracy

To assess intra-observer reproducibility, 15 randomly selected patients (5 skeletal Class II and 10 skeletal Class III) were reanalyzed by the same examiner after an interval of at least two weeks from the original measurements. Repeated analysis included ROI definition, model registration, and volumetric measurements. Intra-class correlation coefficients (ICC) were calculated for Vpre, Vpost, Vres, Vapo, ΔV, and Vnet.

Registration accuracy was assessed using root mean square error (RMSE), mean surface distance (MSD), and 95th percentile distance (P95).

## 3. Results

### 3.1. Qualitative Assessment of the CP Changes After Orthognathic Surgery

**Figure 6:**
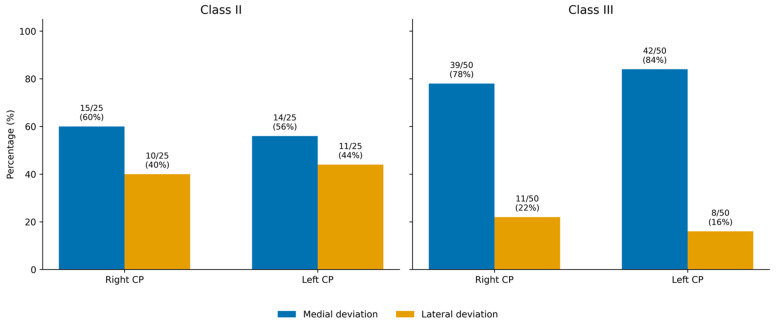
Direction of coronoid process (CP) deviation following orthognathic surgery in skeletal Class II and Class III patients. In both skeletal classes, medial deviation predominated, particularly in Class III. In Class II, a higher proportion of lateral deviations was observed, especially on the left side. Values are presented as absolute counts and percentages (n/N, %) for the right and left CP.

In the qualitative analysis of CP deviation following orthognathic surgery, different distributions of displacement directions were observed depending on the skeletal class. In patients with skeletal Class II, a more heterogeneous pattern of changes was noted, with a slight predominance of medial displacement (60% on the right side and 56% on the left) over lateral displacement (40% and 44%, respectively). In contrast, patients with skeletal Class III demonstrated a clear predominance of medial displacement, observed in 78% of cases on the right side and 84% on the left, with a substantially lower proportion of lateral displacement (22% and 16%). In both groups, the distribution of results was similar between the right and left sides, indicating a generally symmetrical pattern of changes (Figure 6).

As the observed CP deviations were considered to primarily reflect proximal segment positioning, subsequent analyses focused on local postoperative surface changes within the coronoid process. In the skeletal Class II group, a heterogeneous remodeling pattern was observed. Predominantly appositional patterns on the lateral surface were observed in 64% of patients on the right side and 72% on the left side, whereas predominantly appositional patterns on the medial surface were observed in 68% and 48% of patients, respectively. In contrast, predominantly resorptive patterns along the anterior border were observed in 80% of patients on the right side and 60% on the left side. The medial surface on the left side showed a more balanced distribution between appositional and resorptive patterns compared to the right side. These findings indicate a surface-dependent and moderate symmetrical pattern of CP remodeling in skeletal Class II.

**Figure 7:**
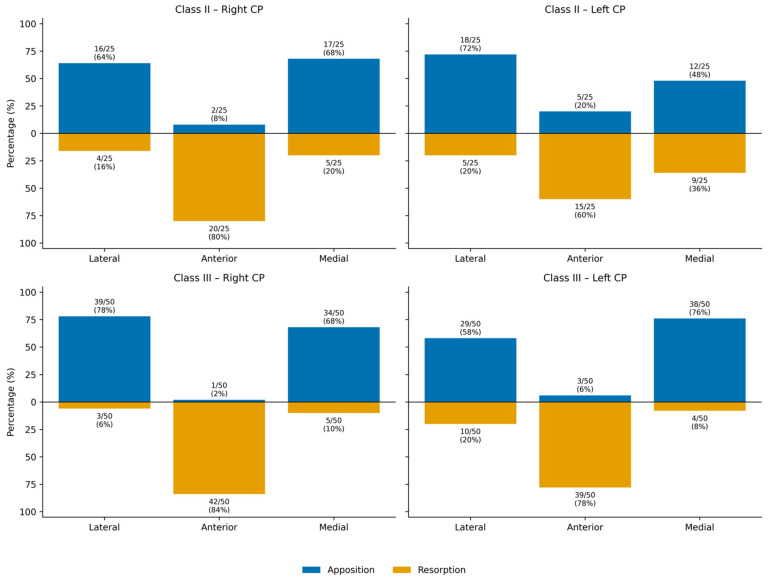
Surface-dependent pattern of coronoid process (CP) remodeling in skeletal Class II and Class III patients. Blue bars represent the percentage of cases with predominantly appositional remodeling patterns, whereas yellow bars represent the percentage of cases exhibiting predominantly resorptive remodeling patterns. In both skeletal classes, appositional patterns were more frequently observed on the lateral and medial surfaces, whereas resorptive patterns were more frequently observed along the anterior border of the coronoid process. In Class III patients, the remodeling pattern appeared more homogeneous and symmetrical between sides, whereas greater inter-side variability was observed in Class II patients. Values are presented as absolute counts and percentages (n/N, %) of cases demonstrating predominance of appositional or resorptive remodeling patterns on the respective coronoid process surface. Negative bars do not represent negative values; they are displayed below the zero line solely to facilitate visual comparison between appositional- and resorptive-dominant patterns.

In the skeletal Class III group, a more pronounced and consistent surface-dependent remodeling pattern was identified. Predominantly appositional remodeling patterns on the lateral surface were observed in 78% of patients on the right side and 58% on the left side, while predominantly appositional remodeling patterns on the medial surface were present in 68% and 76% of patients, respectively. Conversely, predominantly resorptive remodeling patterns along the anterior border were observed in 84% of patients on the right side and 78% on the left side. This distribution was similar on both sides, indicating a symmetrical pattern of changes and a more homogeneous remodeling pattern compared with skeletal Class II (Figure 7).

### 3.2. Quantitative Analysis of Volumetric Changes in the CP

Quantitative analysis assessed differences between preoperative and postoperative parameters (Table 1).

#### 3.2.1. Net Remodeling Balance

In the Class II group, a positive mean net remodeling balance was also observed, more pronounced on the right side (41.8 ± 84.2) than on the left (13.5 ± 45.7). High standard deviations and wide ranges again indicate substantial variability, whereas higher mean and median values (28.6 for the right side and 18.6 for the left) suggest a greater proportion of cases with predominance of appositional processes, particularly on the right side.

In the Class III group, a positive mean net remodeling balance was observed for both the right (15.3 ± 50.0) and left (14.2 ± 50.5) sides. However, high standard deviations and wide ranges indicate considerable inter-individual variability and the coexistence of cases with predominance of either resorption or apposition. Median values (4.2 and 6.6, respectively) suggest that the overall remodeling balance was relatively small in most cases.

#### 3.2.2. Paired Comparisons of Volumetric Remodeling Parameters

In Class II patients, a significant increase in ΔV was observed on the right side (mean difference 6.0%, 95% CI 0.29–11.80%, *p* = 0.042), whereas no statistically significant change was found on the left side (mean difference 1.8%, 95% CI −0.56 to 4.14%, *p* = 0.123).

Regarding the net remodeling balance (Vnet), geometric changes classified as appositional exceeded those classified as resorptive on the right side (mean difference 41.8 mm^3^, 95% CI 7.05–76.54 mm^3^, *p* = 0.042). On the left side, the difference did not reach statistical significance (mean difference 13.5 mm^3^, 95% CI −5.37 to 32.40 mm^3^, *p* = 0.123).

Effect sizes for paired comparisons ranged from small to medium. In the Class II group, a medium effect size was observed for right-sided net remodeling (Cohen’s dz = 0.50), while all other comparisons demonstrated small effects (dz = 0.30–0.43).

In Class III patients, although a small increase in ΔV was observed on both sides, neither comparison reached statistical significance, with a borderline result observed on the right side (*p* = 0.055), while the left side did not show a significant change (mean difference 2.1%, 95% CI −0.32 to 4.58%, *p* = 0.085).

The net remodeling balance (Vnet) indicated that geometric changes classified as appositional exceeded those classified as resorptive on the right side (mean difference 15.3 mm^3^, 95% CI 1.10–29.50 mm^3^, *p* = 0.035), whereas the left side showed a positive but non-significant trend (mean difference 14.2 mm^3^, 95% CI −0.16 to 28.52 mm^3^, *p* = 0.053).

In the Class III group, effect sizes were consistently small across all parameters (dz = 0.24–0.31), indicating a modest magnitude of change despite statistical significance in selected comparisons (Table 2).

#### 3.2.3. Surgical Movement Characteristics

Surgical planning data were available for 41 of 75 patients. In skeletal Class II patients, mean mandibular advancement was 4.9 ± 2.4 mm, whereas Class III patients demonstrated a mean mandibular movement of −0.6 ± 2.7 mm (negative values indicate mandibular setback). Rotational movements showed substantial inter-individual variability in both groups. Detailed movement parameters are presented in Supplementary Table S2.

#### 3.2.4. Correlation Between Surgical Movements and Volumetric Changes

Surgical planning data were available for 41 of 75 patients. Correlation analyses did not demonstrate a significant association between mandibular advancement/setback magnitude and mean relative coronoid process volume change (ΔV) in either skeletal class (Class II: ρ = 0.196, *p* = 0.564; Class III: r = −0.135, *p* = 0.479).

#### 3.2.5. Mixed-Effects Analysis

To account for the non-independence of bilateral measurements obtained from the same patient, a mixed-effects analysis was performed with the patient included as a random effect and side and skeletal class as fixed effects.

For relative CP volume change (ΔV), no significant main effect of side was observed (F = 2.75, *p* = 0.102), indicating that the magnitude of postoperative volume change did not differ significantly between the right and left sides. Likewise, no significant main effect of skeletal class was identified (F = 0.61, *p* = 0.438), suggesting comparable overall volume changes in Class II and Class III patients. The interaction between side and skeletal class did not reach statistical significance (F = 3.21, *p* = 0.077).

A significant random patient effect (*p* < 0.001) indicated substantial inter-individual variability in remodeling response.

For net remodeling balance (Vnet), no significant main effect of side (F = 2.56, *p* = 0.113) or skeletal class (F = 1.14, *p* = 0.290) was found. However, a significant interaction between side and skeletal class was observed (F = 4.22, *p* = 0.044), indicating that side-related differences in remodeling balance varied according to skeletal class. As for ΔV, the random patient effect was significant (*p* < 0.001), confirming substantial variability among individuals (Table 3).

### 3.3. Registration Accuracy

The registration error was low, with RMSE values of 0.102 ± 0.011 mm and 0.096 ± 0.013 mm for the right and left sides, respectively. Corresponding MSD values were 0.097 ± 0.011 mm and 0.092 ± 0.013 mm, while P95 values were 0.134 ± 0.015 mm and 0.127 ± 0.017 mm. The final ICP solution was based on 807 ± 161 and 794 ± 148 inlier points for the right and left sides, respectively. Detailed registration accuracy metrics are presented in Supplementary Table S3.

### 3.4. Intra-Observer Reproducibility

Intra-observer reproducibility was high for all evaluated parameters. ICC values ranged from 0.89 to 0.99 for volumetric measurements. For the principal outcome measures, ICC values reached 0.985 and 0.993 for ΔV and 0.993 and 0.987 for Vnet on the right and left sides, respectively. Complete reproducibility results are presented in Supplementary Table S4.

## 4. Discussion

The aim of the present study was to perform a three-dimensional, semi-automated assessment of early remodeling of the coronoid process following BiMax orthognathic surgery using spatial and volumetric analyses. The results demonstrated that CP remodeling within 6 months after orthognathic treatment is heterogeneous and location-dependent, providing a basis for further investigation of the underlying mechanisms.

The present study demonstrated substantial inter-individual variability in postoperative coronoid process remodeling. Mixed-effects analyses accounting for within-patient correlations did not reveal significant overall effects of side or skeletal class, suggesting that remodeling is driven more strongly by patient-specific factors than by skeletal classification alone. However, a significant interaction between side and skeletal class was identified for net remodeling balance, indicating that side-related remodeling patterns may differ between Class II and Class III patients. Because detailed surgical movement parameters were not available for all patients, the factors underlying these observed differences cannot be determined from the present data and should be interpreted as descriptive rather than causal. These findings highlight the complex and heterogeneous nature of postoperative coronoid process remodeling and underscore the importance of accounting for within-patient correlations when analyzing bilateral anatomical structures.

The proposed association between postoperative coronoid process remodeling and altered temporalis muscle loading is biologically plausible and supported by previous experimental and clinical observations. The relationship between the morphology of the coronoid process and temporalis muscle activity highlights its susceptibility to functional influences. Morphological parameters of this structure have been shown to correlate with electromyographic activity of the TM, confirming its functional nature and dynamic behavior [2]. Moreover, orthognathic treatment leads to significant changes in the activity of masticatory muscles, including the TM, with a tendency toward adaptation during the postoperative period [9]. In this context, the surface-dependent remodeling pattern observed in the present study—characterized by predominant appositional patterns on the lateral and medial surfaces and predominantly resorptive patterns along the anterior border—may be interpreted as a reflection of the redistribution of forces generated by the TM. The muscle inserts on both the lateral and medial surfaces of the coronoid process, and the orientation of its fibers indicates a dominant force vector directed superiorly and posteriorly. Surgical modification of skeletal relationships alters both muscle tension and activation patterns, leading to redistribution of mechanical loading on the CP. Accordingly, the predominance of appositional patterns in areas of muscle attachment may be compatible with an adaptive response, whereas predominantly resorptive patterns along the anterior border may reflect relatively reduced mechanical loading in this region. This muscle-related adaptive mechanism may partly explain why the coronoid process provides information complementary to that obtained from studies of condylar remodeling. The non-uniform distribution of appositional and resorptive remodeling patterns observed in the present study further supports the concept that remodeling tends to occur in specific regions exposed to altered functional demands rather than uniformly throughout the entire structure. Nevertheless, because temporalis muscle activity was not directly assessed, the proposed relationship between remodeling and muscle loading should be regarded as a biologically plausible interpretation rather than direct evidence of causality.

The interpretation of surface differences observed between preoperative and postoperative models requires caution. Because the coronoid process forms part of the proximal mandibular segment, some of the detected changes may reflect a combination of biological adaptation, residual positional changes in the proximal segment following BSSO, segmentation uncertainty, registration error, and mesh-processing effects. Several measures were implemented to reduce these sources of variability, including standardized segmentation, registration restricted to stable ramus regions, automated mesh processing, and reproducibility analysis demonstrating low registration errors and high intra-observer agreement. Nevertheless, the present methodology cannot completely distinguish true biological remodeling from all potential geometric sources of surface variation. Therefore, the terms “apposition” and “resorption” should be interpreted as surface-based remodeling patterns rather than direct histological evidence of bone formation or bone loss.

An important aspect of the present study is the adopted methodology for evaluating remodeling, which was based not on net volumetric change but on the separate assessment of appositional (Vapo) and resorptive (Vres) volumetric differences. This approach enables a more detailed evaluation of remodeling dynamics, as analysis based solely on net balance may obscure the coexistence of substantial opposing patterns of surface change. Normalization of Vapo and Vres to the baseline volume of the CP (Vpre) further facilitates comparison across patients, regardless of anatomical variability. Consequently, this methodology provides a more comprehensive assessment of remodeling by taking into account both the magnitude and balance of imaging-derived volumetric changes.

Quantitative analysis was complemented by a qualitative visualization of the spatial distribution of local surface difference using color-coded heat maps. Although these maps did not provide directional information regarding appositional or resorptive remodeling patterns, they facilitated identification of regions exhibiting greater or lesser morphological change following surgery. Consequently, they should be interpreted as descriptive visualizations of local surface differences rather than quantitative measures of biological remodeling. Such qualitative visualization may help reveal spatially organized patterns of postoperative surface variation that are not readily apparent from global volumetric measurements alone. This approach is consistent with contemporary three-dimensional morphological studies, in which regional surface analysis and model superimposition have been used to characterize localized postoperative structural adaptations. The present analysis was based on three-dimensional reconstruction and precise superimposition of bone models, allowing detailed assessment of the distribution of local surface changes [10].

Another important aspect of the study is the 6-month follow-up period between preoperative and postoperative assessments. Bone remodeling following orthognathic surgery has been shown to represent an adaptive response to displacement of skeletal structures and to begin in the early postoperative phase [11]. The most dynamic changes occur within the first months after surgery [12], whereas the primary adaptive phase of remodeling takes place early, even though the process may continue over a longer period [13]. Therefore, the selected time interval allows for the evaluation of the key phase of remodeling in response to altered biomechanical conditions. In addition, the 6-month follow-up interval corresponded to the standard postoperative protocol used at our institution. At this stage, active treatment is considered completed, and imaging is routinely performed to assess bone union and consolidation at osteotomy sites as well as the absence of inflammatory complications associated with fixation hardware.

The use of artificial intelligence-based tools in three-dimensional image analysis represents a promising methodological development for the study of postoperative morphological changes. Compared to manual techniques, which are more dependent on operator experience, deep learning-based approaches allow for more standardized segmentation, orientation, registration, and analysis of 3D models, reducing the need for extensive manual processing [10,14]. In addition, automation enhances the efficiency of processing large imaging datasets. Nevertheless, quality control of segmentation and superimposition remains essential, particularly when assessing subtle surface changes.

The choice of superimposition technique is critical for the reliability of morphological assessment. The accuracy of voxel-based registration depends on the stability of the reference volume, and remodeling within this region may lead to misregistration and interpretative errors [15]. Furthermore, reduced correspondence of image data between preoperative and postoperative scans may additionally compromise accuracy in postoperative conditions [15]. In the present study, surface-based registration was performed using stable regions of the mandibular ramus, allowing for more reliable assessment of local changes. Selected areas of the proximal mandibular segment have been reported to exhibit relatively limited postoperative remodeling following BSSO and can therefore serve as stable reference regions for superimposition [7].

Postoperative scans contained titanium fixation plates and screws routinely used during orthognathic surgery. Although metallic hardware may generate local imaging artifacts, these structures were not included as separate segmentation classes within the deep learning framework and were therefore excluded from the reconstructed mandibular surface. Following segmentation and mesh reconstruction, fixation sites appeared as localized surface defects corresponding to hardware locations. Importantly, these areas were located outside both the coronoid process region of interest and the predefined reference regions used for model registration. Consequently, their influence on volumetric remodeling analysis was considered limited.

Although no significant association was observed between mandibular advancement/setback magnitude and ΔV within the available subgroup, the incomplete availability of surgical planning records and limited sample size preclude definitive conclusions regarding the influence of surgical movement on coronoid process remodeling. Together with the mixed-effects analysis, these findings suggest that the magnitude of mandibular displacement alone is unlikely to fully explain the variability of postoperative coronoid process remodeling.

Finally, the choice of imaging modality is another important factor influencing the reliability of three-dimensional analyses. In the present study, multidetector computed tomography (MDCT) was used in accordance with the institutional imaging protocol for orthognathic surgery patients. Although cone beam computed tomography (CBCT) is generally associated with lower radiation exposure and high spatial resolution, its gray values are influenced by factors such as field of view, region of interest, acquisition parameters, and image artifacts, which may limit their direct comparability with tissue density measurements obtained using MDCT [16,17]. In contrast, MDCT provides standardized Hounsfield unit measurements and more consistent image characteristics, including superior image uniformity and contrast performance, supporting reliable volumetric and three-dimensional analyses [16,18]. The use of MDCT was also determined by practical considerations, as the field of view available in the CBCT system at our institution was insufficient for comprehensive assessment of all craniofacial structures routinely evaluated during orthognathic surgery planning and postoperative follow-up. Importantly, no additional examinations were performed for research purposes, and all scans were obtained as part of routine clinical care.

Taken together, the present findings indicate that postoperative coronoid process remodeling is characterized by substantial inter-individual variability and a non-uniform spatial distribution of surface changes, while the contribution of surgical movement magnitude and postoperative muscle adaptation remains to be clarified.

## 5. Limitations

The present study has several limitations that should be considered when interpreting the results. First, the retrospective design limited control over patient selection, imaging standardization, and the availability of clinical and surgical data. In particular, detailed information regarding facial asymmetry, deformity subtypes, proximal segment positioning, proximal segment rotation, asymmetry correction, occlusal plane rotation, adjunctive procedures, and complete virtual surgical planning records was not consistently available for all patients. Consequently, the influence of these factors on postoperative coronoid process remodeling could not be comprehensively evaluated, and differences observed between skeletal Class II and Class III patients should be interpreted as descriptive observations rather than evidence of class-specific remodeling mechanisms.

Second, this study assessed remodeling at a single postoperative time point approximately 6 months after surgery. Although this interval corresponds to the period of most active postoperative adaptation and reflects the standard follow-up protocol used at our institution, it does not provide information regarding long-term remodeling changes.

Third, the skeletal Class II group was smaller than the Class III group and demonstrated a markedly unbalanced sex distribution. Therefore, this study was not adequately powered to investigate potential sex-specific remodeling patterns or interactions between sex and skeletal phenotype. Future studies involving larger and more balanced cohorts are warranted.

Several methodological limitations are inherent to image-based shape analysis. Although advanced three-dimensional reconstruction, automated segmentation, standardized registration procedures, and reproducibility analyses were applied, the coronoid process forms part of the proximal mandibular segment, and some of the observed surface differences may reflect a combination of biological adaptation, residual positional changes, segmentation uncertainty, registration error, and mesh-processing effects. Furthermore, validation metrics specific to the coronoid process region were not available because this structure was not treated as a separate segmentation class within the segmentation framework. Consequently, small region-specific segmentation errors cannot be completely excluded. In particular, minor inaccuracies at the coronoid base or along the anterior border may have influenced volumetric estimates in individual cases. Therefore, the reported volumetric parameters should be interpreted within the methodological limitations of image-based three-dimensional analysis.

Another limitation is the lack of direct assessment of functional factors, including temporalis muscle activity and local biomechanical loading. Therefore, the proposed relationship between postoperative remodeling and altered muscle function should be regarded as a biologically plausible interpretation rather than direct evidence of causality.

Finally, postoperative complications, relapse, condylar displacement, and reoperations were not predefined study outcomes and were therefore not systematically recorded or analyzed. Their potential influence on postoperative coronoid process remodeling remains unclear and should be investigated in future prospective studies.

## 6. Conclusions

The present analysis demonstrated that postoperative coronoid process remodeling following orthognathic surgery is characterized by heterogeneous three-dimensional surface changes and substantial inter-individual variability. A consistent surface-dependent remodeling pattern was observed in both skeletal classes, with predominantly appositional remodeling patterns on the lateral and medial surfaces and predominantly resorptive remodeling patterns along the anterior border of the coronoid process.

Quantitative analysis demonstrated an overall positive net remodeling balance, although both positive and negative remodeling balances were observed across individuals. Mixed-effects analyses indicated that individual variability contributed more strongly to remodeling outcomes than consistent effects of skeletal class or side.

The observed remodeling patterns are compatible with adaptive responses to altered postoperative biomechanical conditions; however, the underlying biological mechanisms cannot be established directly from the present data. Future prospective studies incorporating functional muscle assessment, detailed surgical movement parameters, and longer follow-up periods are required to clarify the determinants of postoperative coronoid process remodeling.

## Figures and Tables

**Table 1:** Key CP remodeling parameters 6 months after orthognathic surgery—side-specific analysis.

Parameter	Side	Skeletal Class II (Mean ± SD)	Skeletal Class III (Mean ± SD)
ΔV (%)	R	6.0 ± 13.9	2.5 ± 9.0
	L	1.8 ± 5.7	2.1 ± 8.6
Resorptive volume difference (Vres, mm^3^)	R	54.4 ± 27.1	62.2 ± 38.0
	L	57.5 ± 36.6	56.9 ± 41.9
Appositional volume difference (Vapo, mm^3^)	R	96.2 ± 76.0	77.5 ± 47.7
	L	71.0 ± 38.5	71.1 ± 50.3
Relative resorptive volume difference (%)	R	8.5 ± 5.2	9.0 ± 4.7
	L	8.3 ± 5.5	8.8 ± 5.7
Relative appositional volume difference (%)	R	14.5 ± 12.8	11.5 ± 7.9
	L	10.0 ± 4.9	10.9 ± 7.9
Vnet (mm^3^)	R	41.8 ± 84.2	15.3 ± 50.0
	L	13.5 ± 45.7	14.2 ± 50.5

**Table 2:** Paired comparisons of volumetric remodeling parameters in Class II and Class III patients.

Skeletal Class	Parameter	Side	Vpre Mean ± SD	Vpost Mean ± SD	Mean Diff	95% CI	*p*	Cohen’s dz
Class II	ΔV (%)	R	692 ± 209	734 ± 241	6.0%	0.29–11.80	0.042	0.43
		L	721 ± 230	735 ± 241	1.8%	−0.56–4.14	0.123	0.32
	Vnet (mm^3^)	R	54.4 ± 27.1	96.2 ± 76.0	41.8	7.05–76.54	0.042	0.50
		L	57.5 ± 36.6	71.0 ± 38.5	13.5	−5.37–32.40	0.123	0.30
Class III	ΔV (%)	R	719 ± 279	735 ± 278	2.5%	−0.05–5.07	0.055	0.28
		L	704 ± 298	719 ± 297	2.1%	−0.32–4.58	0.085	0.24
	Vnet (mm^3^)	R	62.2 ± 38.0	77.5 ± 47.7	15.3	1.10–29.50	0.035	0.31
		L	56.9 ± 41.9	71.1 ± 50.3	14.2	−0.16–28.52	0.053	0.28

**Table 3:** Mixed-effects analysis of postoperative coronoid process remodeling.

Outcome	Effect	F	*p*-Value
ΔV	Side	2.75	0.102
	Skeletal class	0.61	0.438
	Side × Skeletal class	3.21	0.077
	Patient (random effect)	3.71	<0.001
Vnet	Side	2.56	0.113
	Skeletal class	1.14	0.290
	Side × Skeletal class	4.22	0.044
	Patient (random effect)	3.26	<0.001

## Data Availability

The datasets generated and/or analyzed during the current study are not publicly available due to patient confidentiality and ethical restrictions. The data are available from the corresponding author upon reasonable request and with permission of the relevant ethics committee.

## Supplementary Materials

The following supporting information can be downloaded at: https://www.mdpi.com/article/10.3390/jcm15134939/s1, Supplementary Materials: Deep Learning-Based Segmentation Framework. Table S1. Demographic and baseline characteristics; Table S2. Surgical movement characteristics; Table S3. Intra-observer reproducibility. Table S4. Registration accuracy.
