# Supplementary material for: Deep Learning-Assisted 3D Analysis of Coronoid Process Changes After Orthognathic Surgery
Source: J Clin Med. 2026 Jun 25;15(13):4939. doi: 10.3390/jcm15134939 (PMC13362027; doi:10.3390/jcm15134939)
Supplement: Supplementary file 1 [file jcm-15-04939-s001.zip › Supplementary Material.pdf]

Supplementary Material

Deep Learning–Based Segmentation Framework

1. Segmentation Framework

Automatic segmentation of anatomical structures was performed using the OrtogOnBlender Startek Edition (OOB-SE) environment. The segmentation module is based on the DentalSegmentator framework, which employs a three-dimensional convolutional neural network derived from the nnU-Net architecture for automated craniofacial CT and CBCT segmentation.

The framework enables automatic extraction of multiple craniofacial structures and was developed to support clinical workflows involving three-dimensional reconstruction, surgical planning, and quantitative morphometric analyses.

2. Training and Validation Datasets

The original DentalSegmentator framework was developed using a dataset comprising 470 CT and CBCT examinations with expert-generated reference segmentations. Model performance was evaluated using independent internal and external test datasets consisting of 133 and 123 scans, respectively.

Reference segmentations were generated and verified by experienced examiners and served as the ground truth during model development and validation.

Importantly, the segmentation model used in the present study was not trained on data originating from the patient cohort included in this investigation, reducing the risk of overfitting and improving generalizability.

3. Network Architecture

The segmentation framework is based on a three-dimensional nnU-Net architecture operating in full-resolution mode. The model employs an encoder–decoder design with automatic optimization of preprocessing, patch size, training parameters, and inference settings according to the nnU-Net framework.

Training was performed using supervised learning with manually generated segmentation masks serving as reference standards.

4. Validation Metrics

Model performance was assessed using standard segmentation accuracy metrics, including Dice Similarity Coefficient (DSC) and Normalized Surface Distance (NSD).

For mandibular structures, Dice coefficients exceeded 0.90, while NSD values approached 0.98, indicating high agreement between automatically generated segmentations and expert-generated reference masks.

These metrics were obtained during the original framework validation and demonstrated robust segmentation performance across both internal and external test datasets.

5. Coronoid Process Segmentation

In the present study, the coronoid process was not segmented as an independent anatomical class. Instead, it constituted part of the mandibular segmentation mask generated by the framework.

Consequently, validation metrics specific to the coronoid process region (e.g., coronoid process–specific Dice coefficient, Hausdorff distance, or volumetric error) were not available. All subsequent analyses of the coronoid process were performed on the segmented mandibular models generated by the validated framework.

6. Study-Specific Workflow

Following automatic segmentation, three-dimensional surface meshes were generated automatically and exported in STL format. No manual modification of mesh geometry was performed prior to model registration and volumetric analysis.

The resulting models served as the basis for qualitative assessment, regional superimposition, volumetric measurements, and evaluation of coronoid process remodeling.
