# Supplementary material for: Deep Learning-Assisted 3D Analysis of Coronoid Process Changes After Orthognathic Surgery
Source: J Clin Med. 2026 Jun 25;15(13):4939. doi: 10.3390/jcm15134939 (PMC13362027; doi:10.3390/jcm15134939)
Supplement: Supplementary file 1 [file jcm-15-04939-s001.zip › Supplementary Table S1.pdf]

Supplementary Table S1. Demographic and baseline characteristics

Characteristic	Class II (n=25)	Class III (n=50)
Age (years), mean ± SD	30.8 ± 7.2	28.5 ± 7.5
Age range (years)	21–41	18–48
Female, n (%)	22 (88.0%)	32 (64.0%)
Male, n (%)	3 (12.0%)	18 (36.0%)
