# Supplementary material for: Deep Learning-Assisted 3D Analysis of Coronoid Process Changes After Orthognathic Surgery
Source: J Clin Med. 2026 Jun 25;15(13):4939. doi: 10.3390/jcm15134939 (PMC13362027; doi:10.3390/jcm15134939)
Supplement: Supplementary file 1 [file jcm-15-04939-s001.zip › Supplementary Table S2.pdf]

Supplementary Table S2. Surgical movement characteristics

Parameter	Class II (n=11)	Class III (n=30)
Mandibular advancement/setback (mm)	4.91 ± 2.39 (0.0–7.0)	−0.57 ± 2.66 (−7.8–5.0)
Mandibular impaction/down-grafting (mm)	−0.05 ± 2.97 (−5.1–5.0)	1.54 ± 1.70 (−2.0–6.1)
Mandibular yaw rotation (°)	0.25 ± 2.35 (−2.8–3.8)	−0.68 ± 2.20 (−6.5–3.7)
Mandibular pitch rotation (°)	−0.65 ± 4.92 (−7.5–6.4)	0.84 ± 3.53 (−12.0–7.5)
Mandibular roll rotation (°)	−0.02 ± 2.30 (−3.8–4.1)	1.18 ± 2.88 (−4.3–8.6)

Surgical planning data were available for 41 of 75 patients and are presented descriptively.
