# Supplementary material for: Deep Learning-Assisted 3D Analysis of Coronoid Process Changes After Orthognathic Surgery
Source: J Clin Med. 2026 Jun 25;15(13):4939. doi: 10.3390/jcm15134939 (PMC13362027; doi:10.3390/jcm15134939)
Supplement: Supplementary file 1 [file jcm-15-04939-s001.zip › Supplementary Table S3.pdf]

Supplementary Table S3. Intra-observer reproducibility and registration accuracy of the proposed workflow based on repeated analysis of 15 randomly selected patients (5 skeletal Class II and 10 skeletal Class III) performed after a minimum interval of two weeks. Reproducibility was assessed using intraclass correlation coefficients (ICC) for preoperative volume (Vpre), postoperative volume (Vpost), resorptive volume difference (Vres), appositional volume difference (Vapo), relative volumetric change (ΔV), and net remodeling balance (Vnet).

Side	R						L					
Parameter	Vpre	Vpost	Vres	Vapo	ΔV	Vnet	Vpre	Vpost	Vres	Vapo	ΔV	Vnet
ICC	0.987	0.988	0.952	0.979	0.985	0.993	0.950	0.947	0.890	0.930	0.993	0.987
