# Supplementary material for: Deep Learning-Assisted 3D Analysis of Coronoid Process Changes After Orthognathic Surgery
Source: J Clin Med. 2026 Jun 25;15(13):4939. doi: 10.3390/jcm15134939 (PMC13362027; doi:10.3390/jcm15134939)
Supplement: Supplementary file 1 [file jcm-15-04939-s001.zip › Supplementary Table S4.pdf]

Supplementary Table S4. Registration accuracy was evaluated using root mean square error (RMSE), mean surface distance (MSD), 95th percentile distance (P95), and the number of inlier points retained in the final ICP solution after outlier rejection. R = right side; L = left side.

**Metric**	**Right side (mean ± SD)**	**Left side (mean ± SD)**
RMSE (mm)	0.102 ± 0.011	0.096 ± 0.013
Mean Surface Distance (mm)	0.097 ± 0.011	0.092 ± 0.013
P95 Distance (mm)	0.134 ± 0.015	0.127 ± 0.017
Inliers (n)	807 ± 161	794 ± 148
